# Anti-*N*-methyl-d-aspartate receptor encephalitis: review of clinical presentation, diagnosis and treatment

**DOI:** 10.1192/pb.bp.113.045518

**Published:** 2015-02

**Authors:** Helen Barry, Susan Byrne, Elizabeth Barrett, Kieran C. Murphy, David R. Cotter

**Affiliations:** 1 Royal College of Surgeons in Ireland, Dublin, Ireland; 2 Trinity College, Dublin; 3 Temple Street Children’s University Hospital, Dublin

## Abstract

Anti-*N*-methyl-d-aspartate (NMDA) receptor encephalitis is a form of encephalitis occurring primarily in women and associated with antibodies against NR1 or NR2 subunits of the NMDA receptor. As a potentially treatable differential for symptoms and signs seen in neurology and psychiatric clinics, clinicians practising across the lifespan should be aware of this form of encephalitis. Common clinical features include auditory and visual hallucinations, delusions, behavioural change (frequently with agitation), impaired consciousness, motor disturbance (ranging from dyskinesia to catatonia), seizures, and autonomic dysfunction. We present a review of the literature on the disorder, including its clinical presentation, differential diagnosis, epidemiology, treatment and prognosis.

Anti-*N*-methyl-d-aspartate (NMDA) receptor encephalitis was first described in 2007 by Dalmau and colleagues,^1^ who identified 12 patients presenting with prominent neuropsychiatric symptoms. All were confirmed to have serum or cerebrospinal fluid (CSF) antibodies to the NMDA receptor.^1^ In a subsequent case series of 100 patients, 77 initially presented to psychiatric services.^2^ Although patients with anti-NMDA receptor encephalitis exhibit high rates of psychiatric disorder, psychiatrists may not be fully aware of the diverse presentation of this disorder. Given the high mortality rate (up to 25%), the likelihood of presentation across the age range and the potential for treatment, a high index of suspicion is warranted by clinicians.

## Epidemiology

Epidemiological studies suggest that anti-NMDA receptor encephalitis may be the most common cause of autoimmune encephalitis after acute demyelinating encephalitis.^3^ While to date there are no estimates as to prevalence rates, more than 500 cases have been reported.^4–6^ Between September 2007 and February 2011, the California Encephalitis Project examined referrals of 761 patients presenting with encephalitis.^7^ Of the cases of identified aetiology, anti-NMDA receptor encephalitis was the leading entity (32 of 79 cases) within the cohort and was identified four times as frequently as herpes simplex – type 1, West Nile virus or varicella zoster virus.^7^ In another study, Steiner and colleagues examined 121 individuals with schizophrenia for diverse NMDA receptor antibodies. Approximately 10% (*n* = 15) were found to be positive for anti-NMDA receptor antibodies, two of whom had the specific immunoglobulin G (IgG) NR1a antibodies of anti-NMDA receptor encephalitis.^8^ Zandi *et al*^9^ observed NMDA receptor serum antibodies in approximately 6% (3 of 46) of patients with first-onset schizophrenia. The true rate of anti-NMDA receptor encephalitis in the population generally and among individuals with psychosis is thus not yet fully clarified.

## Pathophysiology

Encephalitis and psychosis have a long association, with a viral aetiology of psychosis hypothesised as early as the 19th century. The influenza epidemic in the early 20th century led Karl Menninger to conclude that ‘dementia praecox is at least in most instances a somatopsychosis, the psychic manifestations of an Encephalitis’.^10^ While the dopaminergic model of schizophrenia has been the leading neurochemical hypothesis of psychosis for many decades, hypofunction of the NMDA-type glutamate receptors provides an alternative model to understanding the pathogenesis of schizophrenia. It is well described that antagonists of NMDA receptors (e.g. phencyclidine (PCP)) produce a clinical syndrome that closely resembles negative symptoms of schizophrenia and induce neuropsychological and sensory processing deficits that are very similar to those observed in schizophrenia.^11^

The prominent psychotic symptoms, catatonia and indicators of dopaminergic involvement (orofacial dyskinesias) are all consistent with the effects of PCP,^11^ which is well recognised to also replicate many aspects of the presentation of schizophrenia. NMDA receptor antagonists are believed to function by blocking the NMDA receptor in the presynaptic gamma-aminobutyric acid (GABA)-ergic neurons of the thalamus and frontal cortex, leading to a disinhibition of postsynaptic glutamatergic neurons and subsequent glutamatergic and dopaminergic dysregulation in the frontal cortex.

It has been observed that nitroprusside completely abolishes the behavioural effects of PCP in animal models.^12^ Hallak and colleagues^13^ administered a sodium nitroprusside infusion to 20 patients with a diagnosis of schizophrenia, with a resultant rapid improvement in symptoms. They hypothesise that in addition to generating nitric oxide in the brain and increasing cAMP production, nitroprusside may also modulate NMDA receptor activity. In addition, reductions in plasma and brain glycine, D-serine and glutathione levels provide additional mechanisms underlying NMDA dysfunction.^14^

The neuropsychiatric presentation of these cases of anti-NMDA receptor encephalitis thus provides important support for the NMDA receptor hypofunction hypothesis for psychosis.^15,16^ Explanations for the proposed NMDA receptor hypofunction in schizophrenia are varied and may involve altered recycling of NMDA receptors.^17^ However, the possibility that autoantibodies to the NMDA receptor subunits may be causal in psychotic presentations is novel.

Acute psychosis in anti-NMDA receptor encephalitis is associated with serum and CSF IgG antibody titres against the NR1a subunit of the receptor. However, antibodies against different antigens (e.g. the NR2a and NR2b subunits) have been described in cases of limbic encephalitis and systematic lupus erthymatosus,^18,19^ and psychiatric symptoms may not be exclusive to the NR1a subunit. Steiner *et al*^8^ examined the serum of 459 patients admitted with schizophrenia (*n* = 121), major depression (*n* = 70) and borderline personality disorder (*n* = 38) for a variety of antibody subtypes in order to determine whether antibody subtypes overlap with or are distinct from those in anti-NMDA receptor encephalitis. They identified NMDA antibodies in 9.9% of patients with schizophrenia. Diverse NMDA receptor antibodies, including those specifically found in anti-NMDA receptor encephalitis, were found primarily in those with an initial diagnosis of schizophrenia; two patients initially diagnosed with a disorganised or catatonic schizophrenia were subsequently diagnosed with, and treated for, anti-NMDA receptor encephalitis.^8^

Potential causes for the behaviour, learning and memory difficulties in anti-NMDA receptor encephalitis have been postulated by Iizuka and colleagues,^20^ who noted reversible predominant frontotemporal atrophy, an area in which NMDA receptors are present in high density, therefore suggestive of an immunological cause to the atrophy.

Anti-NMDA receptor encephalitis is associated in some cases with ovarian pathology, in particular teratomas. It is considered that the antibodies to the NR1–NR2 subunits of the NMDA subtype of glutamate receptors develop in response to this abnormal tissue.

## Clinical presentation

Presentations can be variable, thus posing a challenge to clinicians in neurology and psychiatry settings. With symptoms and signs ranging from psychosis to mania to catatonia, clinicians may be prompted to consider primary mental health aetiology. Dalmau *et al*^6^ have proposed a staged presentation. Maneta *et al*^5^ summarise these into early, middle and late symptoms, initially involving a prodrome, followed by more overt psychiatric manifestations and later physical symptoms.

Clinicians should be aware that the presentation of anti-NMDA receptor encephalitis includes several characteristic features.

- A non-specific prodrome: in one series of 100 individuals with encephalitis, 86% had headache, low-grade fever or a viral-like illness (headaches, respiratory or gastrointestinal symptoms) in the weeks prior to acute presentation.^2^ In our series of five cases, we identified a prodrome in four, with symptoms including poor concentration, anorexia, insomnia and slurred speech.^6^
- Psychiatric symptoms are prominent: agitation, bizarre and disinhibited behaviour, delusions and auditory and visual hallucinations.^2^ In our series, the psychotic phenomena observed were markedly fragmented in comparison with those typically found in functional psychoses, with delusions being poorly formed and non-systematised.^6^
- Cognitive dysfunction: short-term memory loss can also be a presenting feature, as can concentration difficulties. Formal neuropsychological testing in the presence of psychosis and/or behavioural disturbance may present difficulties in clinical practice.
- Motor dysfunction: in addition to typical epileptic seizures, patients often develop dyskinetic movements, including orofacial dyskinesias (grimacing or lip smacking), which may be mistaken for seizures. These abnormal movements, especially orofacial dyskinesia, may present from an early stage and are often a clue to the diagnosis.
- Autonomic instability: autonomic instability and hypoventilation can also occur (41 of Dalmau’s series^2^ had one or both of these features), as can cardiac dysrhythmias often necessitating intensive care unit management.^2^ Dissociative responses to stimuli during have been noted, including resistance to eye opening while displaying no response to painful stimuli, a combination that may lead to diagnostic confusion.^20,21^
- Association with known pathology: an association with ovarian pathology has also been identified. Dalmau and colleagues reported that in 59% of cases, the diagnosis was associated with ovarian tumours, primarily ovarian teratomas.^2^ However, Irani and colleagues identified tumours in only 26% (9 of 34) of cases.^22^ Children under the age of 18 are unlikely to have an associated tumour.

## Diagnosis

Confirmation of the clinical diagnosis of anti-NMDA receptor encephalitis requires a positive serum or CSF sample screening for antibodies to the NMDA receptor subunit. There is ongoing controversy as to whether serum or CSF is best tested. Dalmau recommends testing of both,^6^ whereas Irani & Vincent,^23^ by contrast, report that serum levels of anti-NMDA receptor antibodies were similar or higher to those of CSF. The clinical symptoms of this disorder correlate well with antibody titre.^2^ The test for anti-NMDA receptor encephalitis, although currently somewhat slow, is relatively cheap, and therefore should be considered in any patient presenting with an acute onset of psychiatric symptoms with atypical features or unusual movements.

CSF abnormalities have been described in approximately 80% of cases and include a mild lymphocyctic pleocytosis, normally or mildly increased protein concentration, and CSF-specific oligoclonal bands.^2,24^

Brain magnetic resonance imaging scans have been reported as normal in 70% of cases.^4^ In the remainder, hyperintensities in a variety of regions may be evident (implicated areas include the hippocampi, cerebellar and cerebral cortex, basal ganglia, brainstem, frontobasal and insular regions).^25^

Typically, electroencephalograms (EEGs) may show non-specific slowing or slow continuous rhythmic activity during the catatonic phase of illness.^26^ An EEG is very helpful if one is trying to distinguish between encephalitis and a primary psychiatric disorder, as the majority of patients (90%) with anti-NMDA receptor encephalitis have evidence of non-specific slowing at some stage during the illness.^4^

While not at present likely to support clinical practice, other investigations have been reviewed. Positron emission tomography has shown variable findings, with some evidence of cortical hypometabolism.^27^ This contrasts with findings from other investigators, suggesting subcortical hypermetabolism.^28^

### Differential diagnosis

The condition may present in the domain of either the neurologist or the psychiatrist, depending on whether psychiatric symptoms precede the neurological features, as is often the case.

#### Neurological

Neurological differential diagnosis tends to include viral encephalitis, cerebral vasculitis or other forms of autoimmune encephalitis and encephalitis lethargica.^29^ Dyskinetic movements may be mistaken for seizure activity or tardive dyskinesia. Patients can also have bizarre stereotypies. Repetitive stereotypies and orofacial dyskinesia can be mistaken for seizures.^30^ The seizure-like dyskinetic movements may also be misdiagnosed as status epilepticus, a diagnosis that is reported in 6% of cases.^30^ Dericioglu and colleagues^30^ report two cases where status epilepticus was suspected but video EEG was indicative of encephalopathy, thus avoiding aggressive treatment with intravenous anaesthetics. Caution therefore is advised in interpreting these movements, unless clarified by video EEG, when status epilepticus is suspected.^30^

#### Psychiatric

Psychiatric differential diagnoses are usually the primary differential in the initial phase of illness. New-onset psychosis is typically recorded in the literature as the most common initial diagnosis because of the presence of delusions, hallucinations and catatonic features. Recent studies explore the possibility that this disorder, or indeed other similar autoimmune conditions, may present with a more typical schizophrenia picture and be responsible for as much as 5–10% of first-onset psychosis.^8,9^ Zandi and colleagues^9^ screened a 46-patient cohort of first-episode psychosis patients in a prospective study for NMDA receptor antibodies and only 2 tested positive. The authors state that there were no clinical features to differentiate these individuals from other individuals with psychosis in the cohort.

Cases of ‘postnatal psychosis’ in association with ovarian pathology that bear remarkable similarity to anti-NMDA receptor encephalitis have also been described.^31^

The presence of rigidity and altered consciousness, which are common in anti-NMDA receptor encephalitis, may also lead to consideration of a diagnosis of neuroleptic malignant syndrome, particularly when antipsychotic medications are prescribed. This may present both a diagnostic dilemma and a management challenge in clinical practice, as these diagnoses are clearly not mutually exclusive.

## Treatment options

It is important to note that treatment must target both the cause and the clinical consequences of the encephalitis (the behavioural and psychotic symptoms). With respect to the former, first-line treatment is immunotherapy, typically corticosteroids, intravenous immunoglobulins or plasma exchange, in addition to the removal of any identified teratomas. Titres are effectively reduced by immunomodulatory treatments, including high-dose steroids, intravenous gamma globulin and plasmaphoresis.

Behavioural disturbance can be a marked obstacle to initiation of treatment, often requiring patients to be sedated for administration of plasma exchange.

Second-line immunosuppression may be necessary using rituximab or cyclophosphamide.

These are often required in individuals who receive a delayed diagnosis or those without a tumour.^26^ Liba *et al*^32^ report use of alemtuzumab in an 8-year-old child with a positive outcome.

Treatment is generally thought to be more effective in patients who have an underlying tumour removed. Cases of ovarian teratomas discovered years after initial onset of symptoms have been described, particularly in patients who experienced a slow recovery.^20^ Peery and colleagues describe a case where oophorectomy was performed despite negative scan results and on postoperative biopsy an occult teratoma was revealed, with subsequent improvement in clinical symptoms.^33^

With respect to the immediate management of behavioural and psychotic symptoms, both typical and atypical, antipsychotics have been utilised. It must be noted that use of antipsychotics can complicate the picture, particularly prior to definite antibody diagnosis. The development of autonomic instability and rigidity may be mistaken for neuroleptic malignant syndrome. In addition, use of corticosteroids may result in confusion with a steroid-induced psychosis. Clonidine, trazadone and benzodiazepines have been used successfully for reversal of sleep disturbance.^34^

Catatonic symptoms are typically treated with benzodiazepine medication. Doses of up to 20–30 mg of lorazepam daily have been used to manage symptoms in catatonia, although little has been published on its efficacy in anti-NMDA receptor encephalitis.^35^ Electroconvulsive therapy (ECT), though the gold standard for treatment of catatonia in the absence of a response to benzodiazepines, is little studied in the area of anti-NMDA receptor encephalitis. Case reports of catatonic symptom response in anti-NMDA receptor encephalitis have been described.^36^ Interestingly, in animal models of ECT action, an elevation of messenger ribonucleic acid (mRNA) of the NMDA subunits NR2A and NR2B has been demonstrated, leading to an up-regulation of the NMDA receptor.^37^

## Prognosis

According to Dalmau’s original case series, approximately 75% of patients with NMDA receptor antibodies recover or have mild sequelae; the other 25% have severe deficits or die.^2^ Subsequent studies have identified a 12–24% risk of relapse.^9,34,35^ Mortality of 7% at 24 months has been noted.^4^ Other studies have noted that approximately 25% of patients at diagnosis give a history of one or more similar symptom episodes in the months preceding diagnosis, indicating a more relapsing and remitting course of illness than initially described.^26^

Titulaer *et al*^4^ in a cohort study of 577 patients noted that first-line immunotherapy resulted in an improvement in 53% of patients in the first 4 weeks of treatment, 97% of whom showed a good outcome at 24 months. In the 47% of patients who did not respond to first-line treatment, those who received second-line immunotherapy (i.e. rituximab, cyclophosphamide or both) had better outcomes than those who continued first-line treatment or received no further immunotherapy.^4^

Several prognostic factors are implicated. With respect to duration of illness and treatment outcome, Finke and colleagues^38^ demonstrated a better cognitive outcome in a small cohort of adult patients with anti-NMDA receptor encephalitis who were treated with immunomodulatory therapy within 3 months of disease’s onset compared with those who were treated at a later stage or not at all. The authors proposed that a delay in treatment may lead to permanent hippocampal damage,^38^ yet the optimal time frame from onset of symptoms to treatment has yet to be determined.

Other identified predictors of outcome include: lower severity of symptoms, not requiring ICU admission, prompt initiation of immunotherapy and tumour removal where present.^4,39^

## Summary and implications to clinical practice

Anti-NMDA receptor encephalitis is a relatively newly identified and potentially treatable cause of psychiatric symptoms in both adults and children. Several hundred cases have been reported since its identification in 2007; however, clinicians may be unaware of developments in this field. It is vital for psychiatrists working across the age spectrum to be aware of this condition and to engage in timely liaison with our neurology colleagues, thus facilitating early screening and diagnosis.

There are a wide range of presenting symptoms and signs. Patients may present with prodromal features, followed by psychiatric and perhaps later physical manifestations. As outlined, anti-NMDA receptor encephalitis is easily diagnosed using a blood or CSF test. This presents the opportunity for early treatment, and a low index of suspicion should be considered for any patient presenting with a constellation of symptoms.

To date, the recommendation for screening has advised testing of those patients, particularly females, with an atypical new-onset presentation of psychosis with motor features. However, recent studies have questioned how readily this disorder is distinguishable from those where patients receive a purely psychiatric psychotic diagnosis.

It is clear that early identification and treatment may have serious prognostic implications. Delay to treatment with immunosuppressive therapy probably results in worsened outcomes, with evidence for permanent hippocampal damage.^38^ Management may prove clinically challenging, from the perspective of treating both the cause and the symptoms. Initiation of antipsychotic treatment is not without risk in these patients and behavioural management may prove challenging.

Anti-NMDA receptor encephalitis is a potentially treatable form of psychiatric illness that is illuminating our understanding of the neuropathophysiology involved in some individuals who present with symptoms of psychosis.

## References

[1] Dalmau J Bataller L Limbic encephalitis: the new cell membrane antigens and a proposal of clinical-immunological classification with therapeutic implications. Neurologia 2007; 22: 526–37. 18000762

[2] Dalmau J Gleichman AJ Hughes EG Rossi JE Peng X Lai M Anti-NMDA-receptor encephalitis: case series and analysis of the effects of antibodies. Lancet Neurol 2008; 7: 1091–8. 18851928 10.1016/S1474-4422(08)70224-2 PMC2607118

[3] Granerod J Ambrose HE Davies NW Clewley JP Walsh AL Morgan D Causes of encephalitis and differences in their clinical presentations in England: a multicentre, population-based prospective study. Lancet Infect Dis 2010; 10: 835–44. 20952256 10.1016/S1473-3099(10)70222-X

[4] Titulaer MJ McCracken L Gabilondo I Armangue T Glaser C Iizuka T Treatment and prognostic factors for long-term outcome in patients with anti-NMDA receptor encephalitis: an observational cohort study. Lancet Neurol 2013; 12: 157–65. 23290630 10.1016/S1474-4422(12)70310-1 PMC3563251

[5] Maneta E Garcia G Psychiatric manifestations of anti-NMDA receptor encephalitis: neurobiological underpinnings and differential diagnostic implications. Psychosomatics 2014; 55: 37–44. 23932531 10.1016/j.psym.2013.06.002

[6] Dalmau J Lancaster E Martinez-Hernandez E Rosenfeld MR Balice-Gordon R Clinical experience and laboratory investigations in patients with anti-NMDAR encephalitis. Lancet Neurol 2011; 10: 63–74. 21163445 10.1016/S1474-4422(10)70253-2 PMC3158385

[7] Gable MS Sheriff H Dalmau J Tilley DH Glaser CA The frequency of autoimmune *N*-methyl-*D*-aspartate receptor encephalitis surpasses that of individual viral etiologies in young individuals enrolled in the California Encephalitis Project. Clin Infect Dis 2012; 54: 899–904. 22281844 10.1093/cid/cir1038 PMC3297648

[8] Steiner J Walter M Glanz W Sarnyai Z Bernstein HG Vielhaber S Increased prevalence of diverse *N*-methyl-*D*-aspartate glutamate receptor antibodies in patients with an initial diagnosis of schizophrenia: specific relevance of IgG NR1a antibodies for distinction from *N*-methyl-*D*-aspartate glutamate receptor encephalitis. JAMA Psychiatry 2013; 70: 271–8. 23344076 10.1001/2013.jamapsychiatry.86

[9] Zandi MS Irani SR Lang B Waters P Jones PB McKenna P Disease-relevant autoantibodies in first episode schizophrenia. J Neurol 2011; 258: 686–8. 20972895 10.1007/s00415-010-5788-9 PMC3065649

[10] Menninger KA Influenza and schizophrenia: an analysis of post-influenza ‘dementia praecox,’ as of 1918, and five years later. Am J Psychiatry 1926; 82: 469–529. 10.1176/ajp.151.6.182 8192197

[11] Baldridge EB Bessen HA Phencyclidine. Emerg Med Clin North Am 1990; 8: 541–50. 2201519

[12] Bujas-Bobanovic M Bird DC Robertson HA Dursun SM Blockade of phencyclidine-induced effects by a nitric oxide donor. Br J Pharmacol 2000; 130: 1005–12. 10882384 10.1038/sj.bjp.0703406 PMC1572164

[13] Hallak JC Maia-de-Oliveira JP Abrao J Evora PR Zuardi AW Crippa JAS Rapid improvement of acute schizophrenia symptoms after intravenous sodium nitroprusside: a randomized, double-blind, placebo-controlled trial. JAMA Psychiatry 2013; 70: 668–76. 23699763 10.1001/jamapsychiatry.2013.1292

[14] Kantrowitz JT Javitt DC N-methyl-d-aspartate (NMDA) receptor dysfunction or dysregulation: the final common pathway on the road to schizophrenia? Brain Res Bull 2010; 83: 108–21. 20417696 10.1016/j.brainresbull.2010.04.006 PMC2941541

[15] Tsai G Yang P Chung LC Lange N Coyle JT D-serine added to antipsychotics for the treatment of schizophrenia. Biol Psychiatry 1998; 44: 1081–9. 9836012 10.1016/s0006-3223(98)00279-0

[16] Stone JM Morrison PD Pilowsky LS Glutamate and dopamine dysregulation in schizophrenia – a synthesis and selective review. J Psychopharmacol 2007; 21: 440–52. 17259207 10.1177/0269881106073126

[17] Lisman JE Coyle JT Green RW Javitt DC Benes FM Heckers S Circuit-based framework for understanding neurotransmitter and risk gene interactions in schizophrenia. Trends Neurosci 2008; 31: 234–42. 18395805 10.1016/j.tins.2008.02.005 PMC2680493

[18] Arinuma Y Yanagida T Hirohata S Association of cerebrospinal fluid anti-NR2 glutamate receptor antibodies with diffuse neuropsychiatric systemic lupus erythematosus. Arthritis Rheum 2008; 58: 1130–5. 18383393 10.1002/art.23399

[19] Mochizuki Y Mizutani T Isozaki E Ohtake T Takahashi Y Acute limbic encephalitis: a new entity? Neurosci Lett 2006; 394: 5–8. 16364542 10.1016/j.neulet.2005.08.070

[20] Iizuka T Sakai F Ide T Monzen T Yoshii S Iigaya M Anti-NMDA receptor encephalitis in Japan: long-term outcome without tumor removal. Neurology 2008; 70: 504–11. 17898324 10.1212/01.wnl.0000278388.90370.c3 PMC2586938

[21] Gonzalez-Valcarcel J Rosenfeld MR Dalmau J Differential diagnosis of encephalitis due to anti-NMDA receptor antibodies. Neurologia 2010; 25: 409–13. 20964986 PMC3101880

[22] Irani SR Bera K Waters P Zuliani L Maxwell S Zandi MS N-methyl-D-aspartate antibody encephalitis: temporal progression of clinical and paraclinical observations in a predominantly non-paraneoplastic disorder of both sexes. Brain 2010; 133: 1655–67. 20511282 10.1093/brain/awq113 PMC2877907

[23] Irani SR Vincent A NMDA receptor antibody encephalitis. Curr Neurol Neurosci Rep 2011; 11: 298–304. 21331529 10.1007/s11910-011-0186-y

[24] Barry H Hardiman O Healy DG Keogan M Moroney J Molnar PP Anti-NMDA receptor encephalitis: an important differential diagnosis in psychosis. Br J Psychiatry 2011; 199: 508–9. 21984802 10.1192/bjp.bp.111.092197

[25] Dalmau J Tuzun E Wu HY Masjuan J Rossi JE Voloschin A Paraneoplastic anti-N-methyl-D-aspartate receptor encephalitis associated with ovarian teratoma. Ann Neurol 2007; 61: 25–36. 17262855 10.1002/ana.21050 PMC2430743

[26] Florance NR Davis RL Lam C Szperka C Zhou L Ahmad S Anti-N-methyl-D-aspartate receptor (NMDAR) encephalitis in children and adolescents. Ann Neurol 2009; 66: 11–8. 19670433 10.1002/ana.21756 PMC2826225

[27] Pillai SC Gill D Webster R Howman-Giles R Dale RC Cortical hypometabolism demonstrated by PET in relapsing NMDA receptor encephalitis. Pediatr Neurol 2010; 43: 217–20. 20691947 10.1016/j.pediatrneurol.2010.04.019

[28] Maeder-Ingvar M Prior JO Irani SR Rey V Vincent A Rossetti AO FDG-PET hyperactivity in basal ganglia correlating with clinical course in anti-NDMA-R antibodies encephalitis. J Neurol Neurosurg Psychiatry 2011; 82: 235–6. 20667855 10.1136/jnnp.2009.198697

[29] Dale RC Irani SR Brilot F Pillai S Webster R Gill D N-methyl-D-aspartate receptor antibodies in pediatric dyskinetic encephalitis lethargica. Ann Neurol 2009; 66: 704–9. 19938173 10.1002/ana.21807

[30] Dericioglu N Vural A Acar P Agayeva N Ismailova V Kurne A Antiepileptic treatment for anti-NMDA receptor encephalitis: the need for video-EEG monitoring. Epileptic Disord 2013; 2: 166–70. 23773862 10.1684/epd.2013.0566

[31] Hopker SW Brockington IF Psychosis following hydatidiform mole in a patient with recurrent puerperal psychosis. Br J Psychiatry 1991; 158: 122–3. 2015434 10.1192/bjp.158.1.122

[32] Liba Z Sebronova V Komarek V Sediva A Sedlacek P Prevalence and treatment of anti-NMDA receptor encephalitis. Lancet Neurol 2013; 12: 424–5. 23602156 10.1016/S1474-4422(13)70070-X

[33] Peery HE Day GS Doja A Xia C Fritzler MJ Foster WG Anti-NMDA receptor encephalitis in children: the disorder, its diagnosis, and treatment. Handb Clin Neurol 2013; 112: 1229–33. 23622333 10.1016/B978-0-444-52910-7.00045-3

[34] Chapman MR Vause HE Anti-NMDA receptor encephalitis: diagnosis, psychiatric presentation, and treatment. Am J Psychiatry 2011; 168: 245–51. 21368306 10.1176/appi.ajp.2010.10020181

[35] Fink M Taylor MA The catatonia syndrome: forgotten but not gone. Arch Gen Psychiatry 2009; 66: 1173–7. 19884605 10.1001/archgenpsychiatry.2009.141

[36] Braakman HM Moers-Hornikx VM Arts BM Hupperts RM Nicolai J Pearls and oysters: electroconvulsive therapy in anti-NMDA receptor encephalitis. Neurology 2011; 75: 44–6. 10.1212/WNL.0b013e3181f11dc1 20819992

[37] Watkins CJ Pei Q Newberry NR Differential effects of electroconvulsive shock on the glutamate receptor mRNAs, for NR2A, NR2B and mGluR5b. Brain Res Mol Brain Res 1998; 61: 108–13. 9795172 10.1016/s0169-328x(98)00211-3

[38] Finke C Kopp UA Pruss H Dalmau J Wandinger KP Ploner CJ Cognitive deficits following anti-NMDA receptor encephalitis. J Neurol Neurosurg Psychiatry 2012; 83: 195–8. 21933952 10.1136/jnnp-2011-300411 PMC3718487

[39] Irani SR Bera K Waters P Zuliani L Maxwell S Zandi MS N-methyl-D-aspartate antibody encephalitis: temporal progression of clinical and paraclinical observations in a predominantly non-paraneoplastic disorder of both sexes. Brain 2010; 133: 1655–67. 20511282 10.1093/brain/awq113 PMC2877907

